# The glass ceiling of endometriosis surgeons is research

**DOI:** 10.52054/FVVO.16.1.011

**Published:** 2024-03-28

**Authors:** P.R. Koninckx, A Ussia, S.W. Guo, E Saridogan

**Affiliations:** Prof em Departments of Obstetrics and Gynecology, in Catholic University Leuven, University Hospital, Gasthuisberg, B-3000 Leuven, Belgium, Radcliffe Hospital University of Oxford, Oxford United Kingdom, Gemelli Hospital Università del Sacro Cuore, Rome, Italy; Gruppo Italo Belga, Villa del Rosario and Gemelli Hospitals Università Cattolica, Rome, Italy; Research Institute, Shanghai OB/GYN Hospital, Fudan University, Shanghai 200011, China; Elizabeth Garrett Anderson Institute for Women’s Health, University College London, United Kingdom

## Introduction

The term ‘glass ceiling’ was coined by Loden (1978) to designate the poorly visible barriers preventing women from being promoted to top jobs in management. Moreover, these poorly visible barriers are hard to remove despite being well-recognised and socially and politically debated for more than 45 years (Lancet, 2018). Later, another poorly visible barrier was added, the glass cliff, pointing to women who tend to be promoted to positions of power during crises or when failure is more likely (Kagan, 2022). Many mechanisms, such as social background, gender, and ethnic origin, contribute to these poorly visible barriers (Kulich et al., 2021).

Poorly visible barriers to women also exist in academic medicine, and the glass ceiling (Tuttle et al., 2020) and the cliff (Krishnan and Szczepura, 2018) were described in 221 and 5 articles, respectively in Pubmed in the last 10 years. The mechanisms in medicine are similar to those in industry, politics, and society in general for women. More recently, leadership competency was added to traditional academic goals such as grants, publications, and citation criteria. In addition, healthcare problems and costs resulted in business as usual being over (Porter and Lee, 2013).

In surgical departments, invisible barriers (Columbus et al., 2020) result in only 7% of surgical department chairs being occupied by women. The androgenic bias in surgical equipment and the risk of injuries for those with smaller hands (Bellini et al., 2022; Koo et al., 2023) is well recognised. Still, instruments continue to be produced in one size only. Although the situation is likely to be similar in obstetrics and gynaecology departments, the consequences of the increased proportion of female doctors, with few male gynaecologists (Karlamangla, 2018) and women becoming the majority in most departments, are not clearly known.

The glass ceiling may not be just related to gender. We should recognise another poorly visible barrier for gynaecological surgeons, making it harder for them to excel in research besides descriptive case series. We will discuss this glass ceiling for research below, using endometriosis surgery as an example.

## Endometriosis research

The history of endometriosis started with clinical observations during surgery and histological examination of specimens (von Rokitansky, 1860; Cullen, 1896; Sampson, 1921). Experiments in humans have been exceptionally rare, except for injections of menstrual blood into the abdominal wall in the 1950s. This would not be acceptable today. Our knowledge of endometriosis was observational, with frequentist statistical analysis of larger data sets and randomised controlled trials (RCT) when made possible by computing power. Bayesian statistics were added only after 2000 because of the calculation power needed (Monte Carlo Markov chain calculations). Our knowledge of endocrinology and the development of immunohistochemistry started (Koninckx et al., 2023) in the early 1970s because of the introduction of radioimmunoassays, the purification of proteins and the generation of specific antibodies.

Research of endometriosis, a disease without an adequate animal model, used to be done by clinicians, mainly surgeons. Even the introduction of radioimmunoassays and histochemistry was performed to a large extent by clinicians spending their spare hours in the laboratories of the departments of obstetrics and gynaecology. This changed dramatically in the 1980s with the introduction and exponential development of molecular biology, requiring a more full-time dedication to research because of the complexity of techniques and the cost of equipment. Consequently, it became increasingly challenging for clinicians to work in departmental laboratories. Especially for surgery-oriented clinicians, this handicap has increased over the years.

The exponentially increasing number of publications made it challenging to read them all. Simultaneously, molecular biology, genetics, epigenetics, proteomics, metabolomics, statistics, and bioinformatics have become increasingly sophisticated, requiring extensive background knowledge and expertise. Laboratory research, being more expensive and requiring more expertise, became organised in larger units based on political priorities and decided by bureaucratic funding bodies. As a result, the combination of part-time research and clinical work became more difficult, especially for surgery-oriented gynaecologists, since the planning of their work was more complicated. The result was a vicious circle, with less clinical input directing research.

## The glass ceiling for endometriosis surgery-oriented gynaecologists

Endometriosis surgery and the endometriosis surgeon changed with the introduction of laparoscopy and deep endometriosis involving the ureter and the bowel. Until the 1970s, endometriosis surgery was performed because of a clinical suspicion but often as an occasional finding during surgery. With the introduction of laparoscopy and knowledge of endocrinology, superficial and cystic ovarian endometriosis surgery became increasingly performed by specialists in reproductive medicine. Most of them had some laboratory background. Subsequently, infertility treatment shifted increasingly to IVF, and laparoscopic surgery of deep endometriosis became more complex, requiring more training. The surgery of the bowel and the ureter caused another shift to surgical sub-specialists, oncologists or abdominal surgeons. Simultaneously, the gap between endometriosis surgery, endocrinology, and research became wider and more glaring.

Endometriosis surgery is poorly suited for research since it does not fit well in evidence-based medicine, emphasising the RCT to avoid bias. However, the many variables in severe endometriosis surgery are difficult to randomise in an RCT, the sample sizes are quite often insufficient for multivariate analysis, and the incidence of complications is too low for statistical evaluation. A multivariate RCT rapidly becomes a logistic nightmare to randomise: a factorial design of 2 factors requires 4 groups, and 3 factors 8 groups. Randomisation is the problem, not statistical power, which is almost similar for each factor to a 1-factor trial with the same number of participants. Endometriosis surgery is inherently multifactorial because of the variability of pathology, such as the severity of at least 3 types (superficial, cystic ovarian and deep), with and without adenomyosis (3*2 groups), with and without pain (3*2*2 groups), the variable localisation of deep endometriosis etc. This variability is prohibitive for a meaningful design and (multivariate) statistical analysis. Moreover, the number of recruitable patients is low since surgery is time-consuming and physically demanding, with limited possibility of delegation (which would add another factor to randomise).

Besides the challenge of sample size, the quality of surgery is difficult to judge. In addition to the primary criterion of achieving the intervention’s goal, such as improvement of pain or quality of life, pregnancy rates or removing the uterus without complications, the outcome of surgery can be assessed by the cost of surgery reflected by the duration (for similar difficulties) and the cost of equipment, by postoperative adhesion formation (increasing with the duration of surgery) and recurrence rates. Besides the multivariate outcome, many endpoints, such as the duration of surgery and complications, cannot be compared since speed does not always reflect quality.

Clinicians, especially endometriosis surgeons, may have less time for reading and writing required for grant applications, research and publications. The time needed for training and the physical stress of surgery makes it difficult to combine it with research, particularly laboratory research.

Finally, it is now becoming apparent that the clinicians’ opinion of a particular treatment is having a significant impact on the recruitment of patients into randomised trials. Surgeons seem to prefer one treatment over the others and are less prepared to randomise patients. Recent examples of the closure of studies in the United Kingdom due to poor recruitment give us concerns about future RCTs in endometriosis and surgical trials in general. The Diamond trial, which was designed to compare surgery for deep endometriosis with medical treatment, was closed by its funders (w3.abdn.ac.uk/hsru/DIAMOND/Public/Public/index.cshtml). Similarly, the closure of the LAVA trial for the same reason as explained by Antoun et al. (2024) in this issue of Facts, Views and Vision may be a sign that the glass ceiling is becoming multi-layered.

## Comments

The research handicap for endometriosis surgeons is poorly recognised, although it is increasing over time. The number of hours available for research has decreased since laparoscopic or robotic surgery requires more hours of training, the working hours have decreased compared with the probably unrealistic working hours of the past, and surgery has become more demanding with the inclusion of bowel and ureter surgery. In addition, the expansion of subspecialties in obstetrics and gynaecology has resulted in fewer surgeries being available for each individual. The laboratory experience of the older generation has become a dream of the past. Physicians were in charge of the research laboratories, with clinical input as a core element. For endometriosis surgeons today, it is increasingly difficult to connect with laboratory research because of the scarcity of available time and the required knowledge, complexity, cost, and personnel needed for laboratory work. Furthermore, trials have become more complicated, often requiring complex statistical analysis.

The invisible barriers for endometriosis surgeons to do research should at least be recognised. It might help us understand why research should be stimulated and why databases of surgical interventions and dedicated statistical help would be a good investment. It is beyond this manuscript to discuss the time needed for training in surgery, getting expertise in laboratory work, database management, and statistical inference, or discussing remuneration and organisation of medicine.

Although keen observers of this glass ceiling, we do not have quick fixes. However, we believe raising awareness may prompt individuals, departments, or societies to devise ways to address this issue. Progress in managing endometriosis that benefits patients is expected to come from the interface of clinicians and research. A multidisciplinary approach and collaboration are likely to be important, but this takes time to build.
